# Minimal residual disease in childhood B Lymphoblastic Leukemia and its correlation with other risk factors

**DOI:** 10.12669/pjms.36.ICON-Suppl.1721

**Published:** 2020-01

**Authors:** Fatima Meraj, Naeem Jabbar, Kishwer Nadeem, Momal Taimoor, Neelum Mansoor

**Affiliations:** 1 Dr. Fatima Meraj, FCPS. Hematology Department, The Indus Hospital, Karachi, Pakistan; 2 Dr. Naeem Jabbar, FCPS. Pediatric Hematology Oncology, The Indus Hospital, Karachi, Pakistan; 3 Dr. Kishwer Nadeem, DCH. Pediatric Hematology Oncology, The Indus Hospital, Karachi, Pakistan; 4 Ms. Momal Taimoor, M.Sc. Hematology Department, The Indus Hospital, Karachi, Pakistan; 5 Dr. Neelum Mansoor, FCPS. Hematology Department, The Indus Hospital, Karachi, Pakistan

**Keywords:** B-lymphoblastic leukemia, Central nervous system, Flow cytometry, Minimal residual disease

## Abstract

**Objective:**

To determine frequency of post induction and post consolidation minimal residual disease (MRD) in pediatric B-lymphoblastic leukemia (B-ALL) patients and its association with clinical risk factors.

**Methods:**

This is a retrospective, cross sectional study carried out at the Indus Hospital on paediatric patients (1-17 years) was performed from May 2015 to January 2018. On day 35, MRD testing was done on bone marrow aspirate using four color flow cytometer with 0.01% cut off. Positive cases were retested at post consolidation. Data was collected for demographics, total leukocyte count (TLC), central nervous system status (CNS), Cytogenetics for BCR-ABL, MLL, TEL-AML by FISH and prophase response then analyzed in association to MRD status.

**Results:**

Out of 362 patients, 133 (37%) were post induction MRD positive, with no statistically significant association to age, gender, TLC, CNS status, prophase response, BCR-ABL and TEL-AML1. However, MLL showed closely significant association (p-value=0.05). Post consolidation, 49 (44%) were MRD positive; age, National cancer institute (NCI) risk groups and CNS status showed statistical significance (p-value <0.05).

**Conclusion:**

Despite high frequency of MRD positivity, significant association is not observed between post induction MRD and risk factors. However, post consolidation MRD has a significant association with NCI risk groups, age and CNS status.

## INTRODUCTION

Minimal Residual Disease (MRD) following therapy is one of the strongest independent prognostic markers for B-lymphoblastic leukemia (B-ALL).^1^ In many studies, MRD has been prognostic at essentially all time points studied i.e. early therapy, during or after induction and early consolidation. Most clinical research on pediatric B-ALL used MRD for risk stratification, which regulates the intensity of post induction therapy.^1^,^2^

Measurement of MRD by flow cytometry is based on the principle that leukemic cells present unusual antigenic patterns that separate them from maturing precursor cells i.e. hematogones. Alternatively, polymerase chain reaction (PCR) DNA sequences specific to leukemia are identified and amplified.^3^,^4^ These techniques reliably detect the presence of at least one in 10,000 leukemic cells in flow cytometry and one in 100,000 in PCR amongst normal bone marrow mononuclear cells.^5^ They are not widely implemented in low middle income countries due to lack of resources and expertise. Flow cytometry has comparatively faster turnaround time, cost effective and less labor intensive. Thus, flow-based MRD assessment has the potential for rapidly identifying patients at increased risk of relapse, allowing for prompt modifications in therapy, including earlier intensification.^6^

Although MRD has major prognostic significance, its co-relation with other prognostic factors has not been thoroughly explored in pediatric ALL patients.^6^,^7^ Some presenting features of ALL show an association to the rate and magnitude by which cytoreduction occurs. However, it has not been established whether MRD has multifaceted associations with other risk factors or whether it is an independent prognostic marker.^8^ With a large hospital database, we studied the association between MRD in the post induction and post consolidation phases of treatment in B ALL patients with risk factors such as National Cancer Institute (NCI) risk classification. NCI, a leading cancer research agency, coordinates the United States national cancer program. NCI risk grouping is based on age and total leukocyte count (TLC) of leukemia patients at the time of diagnosis. Additional risk factors considered were Central Nervous System (CNS) status and cytogenetics.

## METHODS

This is a retrospective, cross sectional study carried out at the Indus Hospital on paediatric patients (1-17 years) with precursor B ALL diagnosed by the haematology laboratory at Ziauddin University Hospital from May 2015 to January 2018. Over the defined study period, data of 437 patients with B ALL was analyzed. Since this is a retrospective analysis carried out on data extracted from hospital database, exemption of ethical clearance was provided by Interactive Research and Development Institutional Review Board (IRD_IRB_2018_04_009).

At presentation, blood samples were collected to determine TLC using the haematological analyzer Coulter LH-500 (Beckman Coulter, USA). Patient’s age and TLC were used to determine the National Cancer Institute (NCI) risk stratification, High risk (HR) constitutes 10 years of age or more with a TLC equal to or greater than 50,000 and Standard Risk (SR) includes patients less than 10 years and with a TLC count less than 50,000. Fluorescence in situ Hybridisation (FISH) was carried out on these patient samples to identify cytogenetic abnormalities BCR-ABL1, MLL rearrangement and TEL-AML 1(ETV6-RUNX1).

A follow up blood cell count and cerebrospinal fluid test through lumbar puncture was conducted on day eight following steroid treatment for the prednisone response and CNS status respectively. CNS status was further classified as CNS1: absence of blasts on cytospin preparation, regardless of the TLC, CNS2: presence of <5/ul TLC count with presence of blasts and CNS3: >5/ul TLC with presence of blasts, traumatic tap with >10 red blood cells/ul and presence of blasts and/or signs of CNS leukemia. After a 7-day prednisone prophase the patients were classified as prednisone-good responders (<1000/μL blasts on day eight) or prednisone-poor responders (>1000/μL blasts on day eight). Based on these risk factors, initial risk stratification was determined and chemotherapy was commenced according to the Berlin-Frankfurt-Munster (BFM) protocol.^9^

On day 35 post induction, MRD using flow cytometry on bone marrow aspirate was conducted. If MRD flow cytometry results were positive on day 35, a post consolidation (Day 52) MRD test was also conducted. Results of post induction and post consolidation MRD were evaluated with respect to the above defined risk factors including age, gender, TLC, NCI risk groups, CNS status and prophase response.

This study included all B-ALL patients with post induction MRD and excluded acute leukemia patients other than BALL phenotype and those who died or left before their MRD assessment or treatment.

### Flow cytometer

Post induction day 35 MRD was detected using a four color FACS Caliber flow cytometer (Becton Dickinson, Biosciences) on mononuclear cells isolated from bone marrow samples. Analysis was carried out using Paint-A-Gate software. A minimum of 500,000 mono nuclear events were acquired. A panel of monoclonal antibodies CD 45-FITC, CD 10-PE, CD 19-PerCP, CD 20-APC, TDT-FITC in two tubes were analysed to identify one in 10,000 (a minimum of 0.01%) of blast cells amongst all mononuclear cells to classify them as MRD positive. Samples were also tested for aberrant immunophenotypic markers CD 13, CD 33, CD 66 and CD 15 if they were identified at the time of diagnosis.

### Statistical analysis

Data was entered on Microsoft Excel and analysed using SPSS version 21.0. Median (IQR) was computed for age and TLC count. Mann-Whitney U test and chi-square test was applied as appropriate to evaluate statistical significance of association of MRD with other risk factors i.e. age and TLC. Chi-square test/Fisher-exact test was applied as appropriate to assess significant association between the cytogenetic markers, gender, CNS 1, 2 and 3, prednisone response with MRD status. A p-value < 0.05 was considered significant.

## RESULTS

Out of 437 B-ALL patients, 362 (82.8%) were tested for post induction MRD and included in the analysis. Seventy-five patients were either lost to follow-up or died during induction. According to our results, 229 (63.3%) of the patients were negative and 133 (36.7%) were positive for MRD at post induction and 49 (44%) were positive at post consolidation. The clinical profile of patients tested in both phases of MRD i.e. post induction and post consolidation are shown in Table-I.

**Table I:** Clinical characteristics of patients.

Clinical characteristics	Post Induction MRD n (%)	Post Consolidation MRD n (%)
*Gender*		
Male	219 (60%)	71 (64%)
Female	143 (40%)	40 (36%)
*Age*		
<10years	258 (71%)	81 (73%)
>10years	104 (29%)	30 (27%)
*Total Leucocyte Count*		
<50x10E9/L	269 (74%)	89 (80%)
50-99.9x10E9/L	47 (13%)	11 (10%)
> 100x10E9/L	46 (13%)	11 (10%)
*NCI*		
Standard Risk	169 (47%)	68 (61%)
High Risk	193 (53%)	43 (39%)
*CNS Status*		
CNS 1	306 (86%)	94 (86%)
CNS 2	26 (7%)	9 (8%)
CNS 3	24 (7%)	6 (6%)

NCI: National cancer institute, CNS: Central nervous system.

Overall, the median age and TLC of patients that were positive for post induction MRD at diagnosis was six years (range 1.5-17) and 15 x10^9^/ul (range 0-656x10^9^/ul) respectively. Of the 362 patients, 258 (71.3%) were below 10 years of age. Similarly, 269 (74.3%) patients had a TLC less than 50x10^9^/L. There were higher number of patients that were categorized as NCI “standard-risk” compared to “high-risk” as shown in Table-II. No significant difference was observed in post induction MRD results in association to all above mentioned variables (*p*-value >0.05).

**Table II:** Association of post induction MRD with clinical and biologic risk factors (n=362)

Risk factors	Criteria	MRD - n=229 (%)	MRD + n=133 (%)	*p*-value
Gender	Male	133 (58)	86 (65)	0.217
	Female	96 (42)	47 (35)	
Age	< 10 years	159 (69)	99 (74)	0.310
	> 10 years	70 (31)	34 (26)	
Total leukocyte count	<50x10E9/L	169 (74)	100 (75)	0.379
	50-99.9x10E9/L	32 (14)	15 (11)	
	> 100x10E9/L	28 (12)	18 (14)	
NCI risk group	SR	115 (50)	54 (41)	0.077
	HR	114 (50)	79 (59)	
CNS Status	CNS 1	196 (86)	110 (85)	0.807
	CNS 2	15 (7)	11 (8)	
	CNS 3	15 (7)	9 (7)	
Prophase response	Good response	172 (82)	102 (82)	0.872
	Poor response	37 (18)	23 (18)	

NCI: National cancer institute, SR: Standard risk, HR: High risk, CNS: Central nervous system

Post induction MRD positivity is apparently higher in NCI HR group (59%) as compare to NCI SR (41%) however statistical significance is not observed. Similarly, results of MRD positivity were evaluated in association to CNS status, higher number of patients found in CNS 1 group but cumulative CNS results showed no significance (p-value=0.807).

Comparative analysis of MRD positive and MRD negative results at post consolidation are shown in Table-III. Post consolidation testing was conducted on patients with positive post induction MRD. Overall, 49 of 111 (44%) patients with residual disease were also positive at post consolidation. Similar to post induction MRD a higher percentage of patients were male in MRD positive group at post consolidation. Clinical and biologic variables at presentation were analyzed in association to post consolidation MRD. No significant difference observed in MRD status with respect to variables including gender, TLC and prophase response (p-value>0.05). In children over 10 years, a higher proportion of MRD positive cases were observed as compared to MRD negative (39% versus 18%, p-value=0.013) and CNS status particularly CNS 2 and CNS 3 (p-value=0.012). Significant difference is observed in post consolidation MRD and NCI risk group i.e. SR and HR groups showed 45% and 55% MRD positivity respectively (p-value=0.002).

**Table III:** Association of post consolidation MRD with clinical and biologic risk factors (n=111).

Risk factors	Criteria	MRD - n=62 (%)	MRD + n=49(%)	*p*-value
Gender	Male	39 (63)	32 (65)	0.793
	Female	23(37)	17 (35)	
Age	< 10 years	51 (82)	30 (61)	0.013*
	> 10 years	11 (18)	19 (39)	
Total leukocyte count	<50x10E9/L	53 (85)	36 (74)	0.263
	50-99.9x10E9/L	5 (8)	6 (12)	
	> 100x10E9/L	4 (6)	7 (14)	
NCI risk group	SR	46 (74)	22 (45)	0.002*
	HR	16 (26)	27 (55)	
CNS Status	CNS 1	58 (94)	36 (73)	0.012*
	CNS 2	2 (3)	9 (18)	
	CNS 3	2 (3)	4 (8)	
Prophase response	Good response	46 (79)	40 (85)	0.443
	Poor response	12 (21)	7 (15)	

* : Significant value, NCI: National cancer institute, SR: Standard risk, HR: High risk, CNS: Central nervous system.

**Fig.1:**
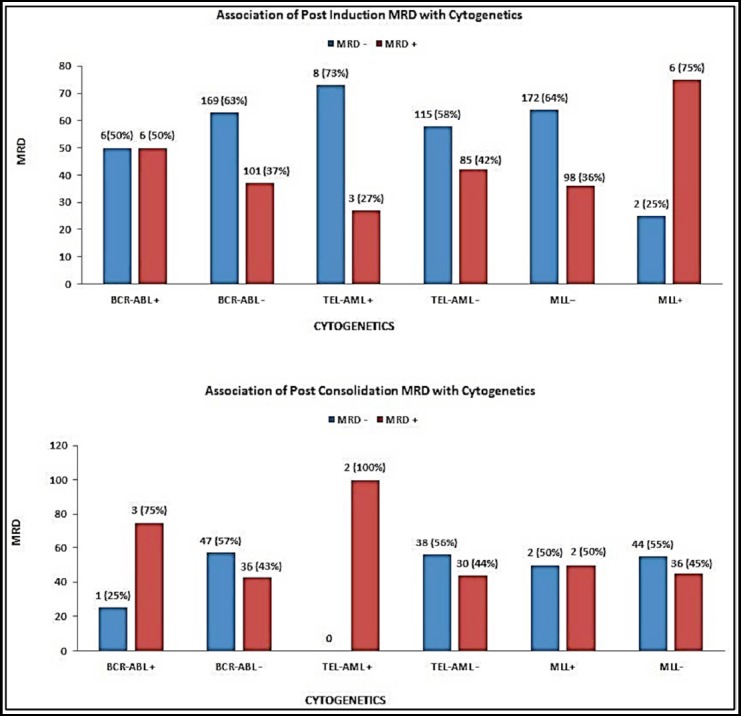
Association of Post Induction MRD and Post Consolidation MRD with Cytogenetics

Additionally, to check the presence of cytogenetic abnormalities in association to MRD, FISH data was also analysed for both post induction and post consolidation MRD results. A higher percentage of the patients were MRD negative in association with each of the cytogenetics as shown in Fig.1. However, a 50% distribution in post induction MRD status was observed when the patients were positive for BCR-ABL. Close to significant difference in post induction MRD status was observed when analyzed with respect to MLL positive cases (p-value=0.06). No significance is observed with any cytogenetic when analyzed in association to post consolidation MRD status. Fig.1.

## DISCUSSION

To the best of our knowledge, this is the first study on MRD conducted in Pakistan using flow cytometry with a very good sample size and sensitive diagnostic technique, it reflects treatment response in terms of MRD in our population and highlights the importance of monitoring at cellular level using sensitive techniques. Studies of MRD considerably enhance the precision of risk assessment, a task of paramount importance in the treatment of childhood ALL.^5^ A study conducted by Neale et al. compared the detection efficacy of the two techniques i.e. PCR and flow cytometry, both techniques gave concordant MRD-positive or MRD-negative results in (96.7%) of the samples.^5^ MRD detection for B-ALL by flow cytometry is well described in literature.^4^,^10^ However, in low middle income countries, traditional morphological remission criteria is widely used to monitor residual disease which is quite subjective and has limited sensitivity. The expensive nature and dearth of training of flow cytometry has made it a scarce facility in low middle income countries.^11^ While it has been established as an attractive technique across the world, there is a scarce usage of flow cytometry to detect MRD in Pakistan and this is the first study which demonstrates provocative results that MRD assessment in isolation and/or in association to other clinical features helps in risk stratification, treatment modification as well as monitoring.^12^

Post induction and consolidation MRD have been established as a primary prognostic factor in ALL.^13^ The frequency of MRD positivity of our patients was 37% for post induction and 44% for post consolidation which is relatively higher than other reported studies using a similar protocol.^6^,^7^,^14^ A Children’s Oncology Group study reported by Borowitz et al. showed 28.6% end induction MRD positivity.^15^ This could be due to the fact that we are catering to a specific demographic that is unable to sustain a balanced diet and environment due to economic constraints. Moreover, delayed presentation, high disease burden, limited supportive care and compliance issues are possible explanations but further research should be carried out to determine the cause behind a relatively higher MRD positivity than internationally published data.

Presenting clinical features like age, TLC and prophase response are directly related to the extent of initial cytoreduction and used to gauge the effectiveness of therapy. We compared the status of post induction MRD with respect to clinical and biological risk factors at the time of presentation and no significant difference is observed (p-value >0.05). Borowitz et al. reported association of end induction MRD with NCI HR which is not observed in our study.^15^ Dario Campana reported significant frequency of residual disease in infants and children > 10years of age, NCI HR and presence of BCR-ABL which is contrary to our results.^16^ We cannot comment on MRD frequency in infants as patients less than one year of age were excluded from the study. Similarly, prophase poor response is also reported to have strong association with residual disease and early relapse however such predilection is not observed in our data.^16^ In our study, apparently, MRD positivity at post induction was more in NCI HR however number of patients are also higher in HR group as delayed clinical presentation is common in our clinical settings. We would recommend multicenter research to evaluate association of these clinical features with post induction MRD.

Likewise, cytogenetic abnormalities characteristic of B-ALL largely determine the biology of the disease, affect prognosis, and guide therapy.^7^ Presence of Ph+ BCR-ABL and MLL rearrangement are considered to have poor outcome. On the other hand, TEL-AML1 (ETV6-RUNX1) was established to be a good prognostic indicator. Our results demonstrated that TEL-AML1 positivity had a 72.7% chance of no residual disease maintaining its position to have good outcome. In contrast to literature no remarkable association is found in post induction MRD positivity and presence of Philadelphia chromosome.^1^ Published studies have shown BCR-ABL to be a bad prognostic factor with a higher chance of relapse and MRD positivity with its presence but our results are not supportive.^17^ MLL rearrangement showed the closest correlation to MRD with a higher chance of MRD being positive with positive MLL gene rearrangement and vice versa (p-value=0.056).^18^ One limitation that this result poses is that a total of eight samples were positive for MLL gene and that is very low number. However six out of eight were MRD positive. These cytogenetic abnormalities that were tested had no association with post consolidation MRD status.

Furthermore, CNS involvement at diagnosis is associated with adverse prognostic features but further research into the biologic basis of inferior response is essential. Individual host response to chemotherapeutic agents is influenced by genetic polymorphisms in drug transporters and metabolizing enzymes.^8^ Our results are consistent with literature and shows no significant difference in bone marrow MRD level after induction when comparing the CNS status in patients, this can also be that the presence of CNS disease does not indicate a less chemo sensitive leukemia. However, among the patients evaluated for post induction MRD levels, the CNS1 group and the patients with CNS involvement (CNS 3) are not completely comparable when it comes to induction therapy since patients with CNS involvement received additional therapy.^19^

MRD is a context-dependent variable with different prognostic meanings at different time points. Very early conversion to MRD negativity indicates an excellent prognosis, whereas MRD negativity at a late time point is still associated with a considerable relapse rate. In a study by Vendetti et al., post consolidation MRD status emerged as an independent variable that was significantly associated with a high frequency of relapse (*p* = 0.001). Thus confirming the highly predictive role of MRD status at the end of consolidation.^20^ Our study revealed a significantly higher MRD positivity particularly at post consolidation phase in contrast to reported literature that is self-explanatory of inferior survival outcome in comparison to rest of world.^6^,^7^ Our results showed significant difference in post consolidation MRD status in association to age, NCI and CNS status (p-value<0.05).

At present it seems advisable to evaluate MRD results in combination with known prognostic variables though according to our study association with other variables is weak or absent at post induction MRD. Conversely there are studies which illustrate that MRD enhance the informative utility of these variables and are useful in comprehensive risk assignment to the patients.^21^ Therefore, we cannot exclude the significance of clinical features in distinguishing the patients who require intense therapy from those who require less intense therapy that needs to be proved by similar studies with larger sample size. Our study has relatively higher MRD positivity and further multi-center studies should be conducted where patients MRD status and relapse is monitored till end of treatment and subsequently evaluated with other risk factors to present a more accurate prognostic potential.

## CONCLUSION

Detection of MRD using flow cytometry is a useful approach for predicting MRD in patients with B ALL. Our study shows a relatively higher positivity of MRD at post induction and post consolidation phases in our population. Significant association is not observed between post induction MRD and risk factors however post consolidation MRD has a significant association with NCI risk groups, age and CNS status.
